# Bipolar Switching Characteristics of Transparent WO_X_-Based RRAM for Synaptic Application and Neuromorphic Engineering

**DOI:** 10.3390/ma15207185

**Published:** 2022-10-15

**Authors:** Jihyung Kim, Jongmin Park, Sungjun Kim

**Affiliations:** Division of Electronics and Electrical Engineering, Dongguk University, Seoul 04620, Korea

**Keywords:** transparent resistive random-access memory, synapse device, neuromorphic engineering, tungsten oxide, indium tin oxide

## Abstract

In this work, we evaluate the resistive switching (RS) and synaptic characteristics of a fully transparent resistive random-access memory (T-RRAM) device based on indium-tin-oxide (ITO) electrodes. Here, we fabricated ITO/WO_X_/ITO capacitor structure and incorporated DC-sputtered WO_X_ as the switching layer between the two ITO electrodes. The device shows approximately 77% (including the glass substrate) of optical transmittance in visible light and exhibits reliable bipolar switching behavior. The current-voltage (I–V) curve is divided into two types: partial and full curves affected by the magnitude of the positive voltage during the reset process. In the partial curve, we confirmed that the retention could be maintained for more than 10^4^ s and the endurance for more than 300 cycles could be stably secured. The switching mechanism based on the formation/rupture of the filament is further explained through the extra oxygen vacancies provided by the ITO electrodes. Finally, we examined the responsive potentiation and depression to check the synaptic characteristics of the device. We believe that the transparent WO_X_-based RRAM could be a milestone for neuromorphic devices as well as future non-volatile transparent memory.

## 1. Introduction

The development of invisible electronic systems provides a great opportunity for the semiconductor market and urges the advancement of device application. The attempts to incorporate various transparent electronic devices are being made in various fields such as TFTs [1], OLED displays [2,3], artificial skins [4], chemical sensors [5], and solar cells [6,7]. In order accomplish this successfully, it is necessary to fabricate a highly reliable transparent memory for information storage. The non-volatile memory, one of the critical components, needs to meet the demand for high optical transmittance for transparent integrated circuit application. Among next-generation non-volatile memories, RRAM can meet the standards of high transparency and is a promising candidate due to its simple metal-insulator-metal (MIM) structure, low power consumption, high-density scalability, excellent memory performance (endurance and retention), fast data processing, and multi-level characteristics [8,9,10,11,12,13,14,15,16,17,18].

RS behavior is usually classified into unipolar and bipolar, depending on the voltage polarity of the write and erase process. While unipolar switching indicates the change in resistance occurred in one voltage direction, bipolar switching refers to both voltage directions of the write and erase operation. In recent times, the coexistence of both switching behaviors in a single metal-oxide material such as NiO_X_ [19], CoO_X_ [20], TiO_X_ [21], WO_X_ [22], ZnO_X_ [23,24], and BaSrTiO_3_ [25] have received much attention. It is widely known that both unipolar and bipolar RS typically depend on the formation and rupture of conductive filaments in the oxide switching layer [26].

T-RRAM differs from conventional RRAM because it uses transparent materials as the electrodes and switching layers; examples include aluminum-doped zinc oxide (AZO) and ITO [27,28,29,30]. The high optical transmittance and high electrical conductivity make them suitable for applications in various transparent electrode fabrication. For the development of high optical transparency in memory devices, switching materials with wide band gaps is essential. WO_X_ has a wide bandgap of about ~3.01 eV [30,31] which is suitable for acting as a switching layer in the memory device. Moreover, it has numerous advantages such as high compatibility with the back-end-of-line (BEOL) process in complementary metal-oxide-semiconductor (CMOS) technology, easy fabrication, and high thermal stability [32]. 

In this study, we investigated the resistive properties of T-RRAM based on the capacitor structure of ITO/WO_X_/ITO. First, we checked the atomic composition of each thin film and the optical transmittance using analysis equipment. After giving the voltage to confirm the memory function, no forming process was required; the same bipolar switching but different RS behavior were observed in our device in accordance with the first reset process. The effect of the applied voltage on the formation and rupture of the conductive filaments was expressed as a schematic image and its verification as the synaptic device was confirmed through reproducible potentiation and depression.

## 2. Materials and Methods

The ITO/WO_X_/ITO device was fabricated in the following process: ITO, which was deposited on a commercially available glass substrate, was prepared for the bottom electrode (BE). The substrate was cleaned using acetone, isopropyl alcohol (IPA), and deionized (DI) water under ultrasonication for 5 min each. The switching layer of WO_X_ deposited about 15 nm at room temperature. The 4-inch tungsten metal target was sputtered through pulsed DC power of 0.2 kW and a frequency of 25 kHz. The process pressure in the main chamber was maintained at 1 mTorr and the gas flow rate was set to 8 sccm for Ar gas and 12 sccm for O_2_ gas. Finally, the ITO top electrode (TE) was patterned with a circular shadow mask (100 µm in diameter) and deposited by an E-beam evaporator with a thickness of 100 nm. 

The cross-sectional transmission electron microscope (TEM) examined the microstructure and thickness analysis of the device. The X-ray photoelectron spectroscopy (XPS) analyzed the atomic composition in the depth profile. The UV-visible scanning spectrophotometer was used to measure the light transmittance ranging from 200 nm to 900 nm. The Keithley 4200-SCS semiconductor parameter analyzer and the 4225-PMU ultrafast pulse modules were used to evaluate the electrical characteristics of the device. 

## 3. Results and Discussion

**Figure 1:**
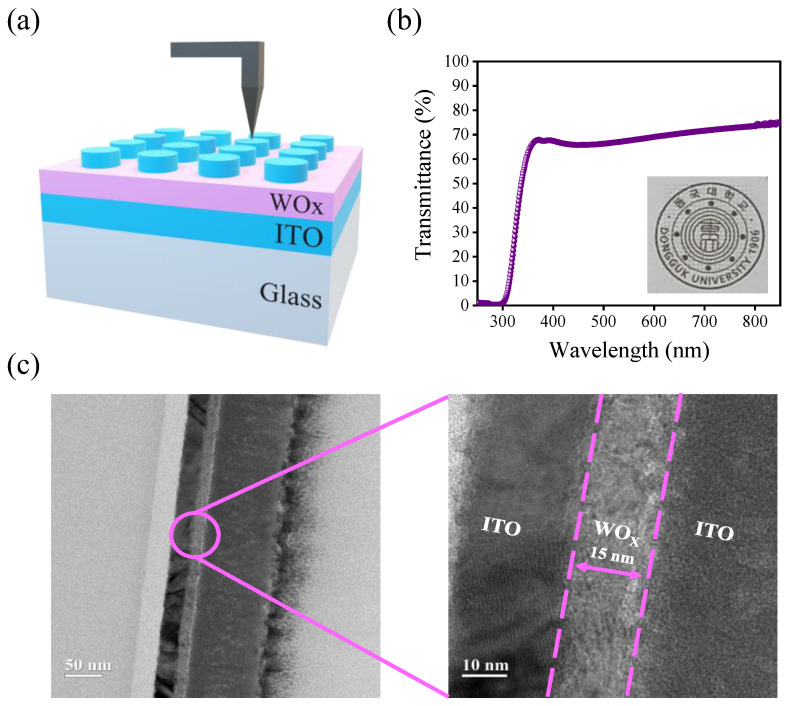
(**a**) Schematic image of ITO/WO_x_/ITO device. (**b**) Optical transmittance spectrum measured using a spectrophotometer in the visible wavelength region. Inset image shows the transparency of the fabricated device. (**c**) Low and high magnification imaged by TEM.

Figure 1a shows the schematic image of the device structure during the measurement of electrical characteristics by setting the BE into the ground bias. The TE was contacted with the probe tip to apply the voltage. As shown in Figure 1b, the transmittance in the visible region was confirmed through a UV-visible scanning spectrophotometer showing 77% transparency at a wavelength of 400 nm to 800 nm. The logo (the inset image of Figure 1b, meaning Dongguk University in Korean, Seoul, Korea) located below the device was clearly seen without any distortion or refraction. The presence and clear distinction of each layer can be confirmed through the TEM image of ITO/WO_X_/ITO; each layer is separated by the dotted lines as presented in Figure 1c. It shows the 15 nm thickness of WO_X_ film deposited in an amorphous state due to the sputtering process.

**Figure 2:**
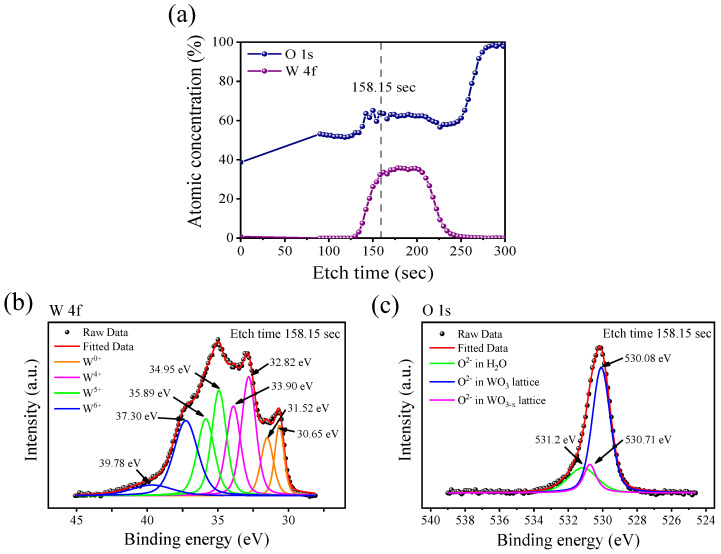
(**a**) Atomic concentration in accordance with the etch time. Only the information about W 4f and O 1s is specified. (**b**) Four different doublets of W 4f core level spectra in the etch time of 158.15 s. (**c**) O 1s core level spectra in the etch time of 158.15 s.

In order to understand the elemental composition profile and chemical structure of the WO_X_ switching layer, we analyzed the XPS data. Figure 2a shows the results of examined atoms in accordance with the etch time; only the core levels of W 4f and O 1s were specified. Since the etching starts from the TE to the BE, the black dotted line of 158.15 s indicates the WO_X_ layer located just below the ITO electrode. The binding energy was re-scaled with respect to the C 1s signal (284.8 eV) corresponding to the native carbon existing on the sample surface. The W 4f spectra can be deconvoluted into two doublets, as shown in Figure 2b. The binding energy of the blue line at 39.78 eV (W 4f_5/2_) and 37.30 eV (W 4f_7/2_) corresponds to the W^6+^ oxidation state. Similarly, the W^5+^ oxidation state and the W^4+^ oxidation state correspond to the green and pink doublets, respectively. The final doublet of the orange line shows the binding energy at 31.52 eV (W 4f_5/2_) and 30.65 eV (W 4f_7/2_), which indicates the W^0+^ metallic state. The O 1s core-level spectra can be divided into three components as shown in Figure 2c. The first component has a binding energy of 530.08 eV that is assigned to the oxygen atoms in stoichiometric WO_3_. The second component observed at 530.71 eV has been attributed to the oxygen atoms in the sub-stoichiometric WO_3–X._ The last component is at 531.2 eV which was the oxygen in water molecules pertaining to the moisture adsorbed on the sample surface. 

**Figure 3:**
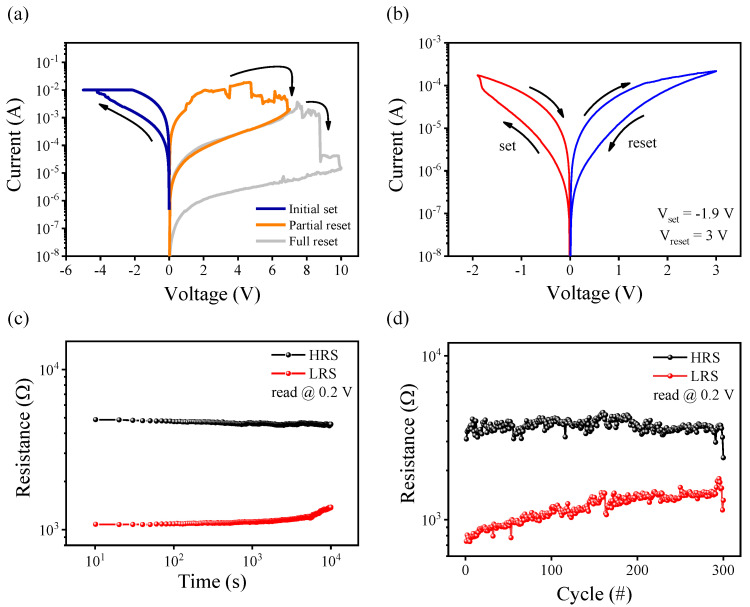
Electrical characteristics of ITO/WO_X_/ITO device. (**a**) First voltage sweep determining the type of I–V curve according to the reset process. (**b**) Gradual analog switching achieved from the partial reset. (**c**) Retention, read at the voltage of 0.2 V at room temperature for over 10^4^ s. (**d**) Endurance characteristics evaluated through DC voltage cycles.

We investigated the bipolar RS behaviors and they could be divided into two types according to the reset process, as mentioned above. In Figure 3a, the compliance current of 10 mA was set to prevent the hard breakdown and ensure stable switching. After reaching the I_CC_ with the first linear voltage sweep of -5 V, the reset voltage of 7 ± 0.5 V or the reset voltage of 10 ± 0.5 V decided the different types of I–V curves. The device-to- device leakage current for the positive set and the negative set is illustrated in Figure S1. The flow of high current in the pristine state indicates that a sufficient amount of oxygen vacancies is located inside WO_X_ and the device does not require a forming process. Figure 3b shows the gradual analog switching achieved after the partial reset, which is different from the abrupt digital switching induced after the full reset with a voltage of about 10 V. The abrupt switching curve can be observed in Figure S2. The high resistance state (HRS) and low resistance state (LRS) were read at 0.2 V for both retention and endurance measurements. The retention test was performed and each resistance state was found to be constant at room temperature for over 10^4^ s as shown in Figure 3c. Figure 3d displays the DC endurance test with stable and uniform switching behavior for over 300 cycles.

**Figure 4:**
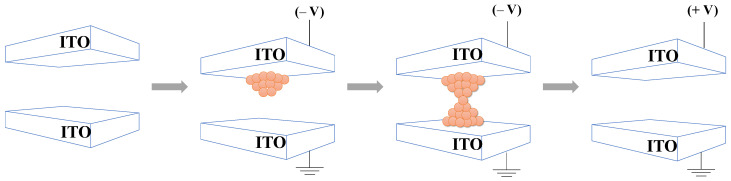
Schematic diagram of switching operation in valence change memory. Partial reset curve occurred at the voltage of 7 V and full reset curve occurred at the voltage of 10 V.

We studied the switching mechanism and sketched the schematic diagram during the initial switching referring to the I–V curve in Figure 3a. As shown in Figure 4, oxygen atoms oxidize when TE is negatively biased, then oxygen vacancies (V_0_) migrate to the TE/WO_X_ interface. Both ITO and WO_X_ act as n-type semiconductors; therefore, the V_0_ can play the role of n-type dopants. A negative voltage applied on the TE attracts V_0_ to the TE/WO_X_ interface forming the conductive paths, known as the set operation. Moreover, schematic diagrams of the energy band alignment of the ITO/WO_X_/ITO device are illustrated in Figure S3. The band alignment of the device is shown in its initial state with zero bias. The work function of ITO is 4.7 eV and the electron affinity and bandgap of WO_X_ are 3.33 eV and 2.8 eV, respectively. The thickness of the insulator and the bandgap could affect the switching properties. 

Figure 3a shows the partial reset curve and full reset curve. It is widely known that the resistance in the “ON” state is related to the size of conductive filaments. On the other hand, the “OFF” state resistance depends on the remaining part of the unruptured filaments [32]. In our device, we can control the V_reset_ to achieve gradual analog resistive switching for synaptic devices.

**Figure 5:**
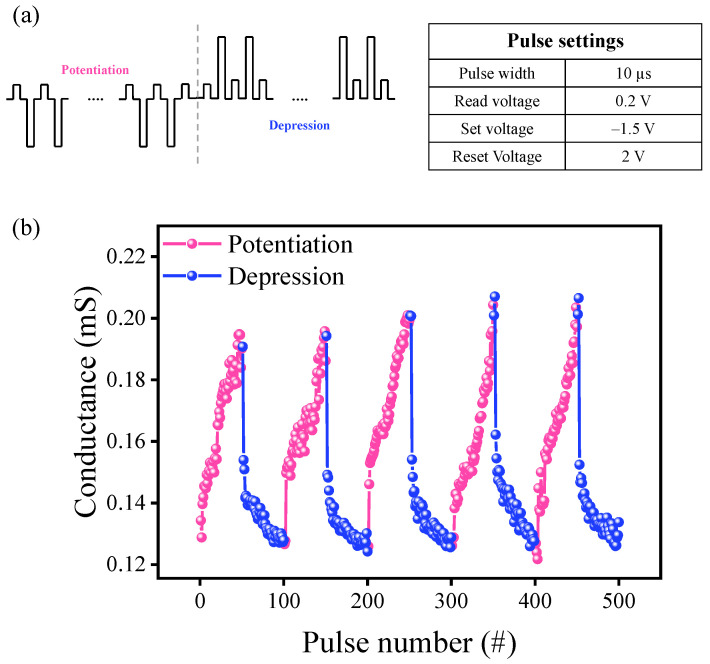
(**a**) Pulse settings used for the potentiation and depression measurement. (**b**) Conductance changes during the 500 consecutive identical pulses.

Figure S4 shows a multi-level cell (MLC) by controlling reset voltage. Moreover, we demonstrate more conductance states by pulse response. Figure 5a shows the pulse settings composed of identical pulses and specifications written in the table. A low read voltage of 0.2 V was used so as not to affect the conductance change during the read process. The set and reset voltage during the pulse measurements were −1.5 V and 2 V, respectively. Figure 5b shows the transient characteristics of the set and reset process. We demonstrated 5 cycles of long-term potentiation (LTP) and long-term depression (LTD) and found no or small conductance change after applying each identical pulse [33]. In other words, the conductance range for potentiation and depression was well-maintained through the identical pulses during the repetitive operation. The confirmation of conductance modulation during the potentiation and depression from these characteristics shows that the ITO/WO_X_/ITO device is suitable for application in neuromorphic engineering and synapse devices. 

## 4. Conclusions

In summary, the transparent ITO/WO_X_/ITO memristor was investigated to mimic the biological synaptic functions for neuromorphic engineering. Two different types of switching behavior were obtained by controlling the voltage during the first reset process. Due to the linear synaptic property, we focused on gradual analog switching which can be obtained by applying partial reset. Long retention above 10^4^ s and stable cycling endurance for 300 cycles were observed. We also evaluated the LTP/LTD characteristics which indicated a resemblance to the neurotransmitters in the biological synapse. We consider the T-RRAM device in this work as promising research for indicating biological synaptic features and electrical memory characteristics.

## Data Availability

Not applicable.

## Supplementary Materials

The following supporting information can be downloaded at: https://www.mdpi.com/article/10.3390/ma15207185/s1, Figure S1: Device-to-device leakage current curves for positive set and negative set; Figure S2: Abrupt switching curve after applying large voltage for a full reset; Figure S3: Schematic diagram of band alignment of ITO and WO_X_; Figure S4: MLC by DC sweep by controlling reset voltage.

## References

[1] Masuda S., Kitamura K., Okumura Y., Miyatake S., Tabata H., Kawai T. (2003). Transparent thin film transistors using ZnO as an active channel layer and their electrical properties. J. Appl. Phys..

[2] Geffroy B., Le Roy P., Prat C. (2012). Organic light-emitting diode (OLED) technology: Materials, devices and display technologies. Polym. Int..

[3] Park S.H.K., Ryu M., Hwang C.S., Yang S., Byun C., Lee J.I., Shin J.H., Yoon S.M., Chu H.Y., Cho K.I. (2008). Transparent ZnO thin film transistor for the application of high aperture ratio bottom emission AM-OLED display. SID Sym. Dig. Tech. Pap..

[4] Ramuz M., Tee B.C.K., Tok J.B.H., Bao Z. (2012). Transparent, optical, pressure-sensitive artificial skin for large-area stretchable electronics. Adv. Mater..

[5] Moon H.G., Shim Y.S., Kim D.H., Jeong H.Y., Jeong M., Jung J.Y., Han S.M., Kim J.K., Kim J.S., Park H.H. (2012). Self-activated ultrahigh chemosensitivity of oxide thin film nanostructures for transparent sensors. Sci. Rep..

[6] Kim S., Patel M., Nguyen T.T., Yi J., Wong C.P., Kim J. (2020). Si-embedded metal oxide transparent solar cells. Nano Energy.

[7] Ruiz-Perona A., Sánchez Y., Guc M., Khelifi S., Kodalle T., Placidi M., Merino J.M., Leon M., Caballero R. (2020). Effect of Na and the back contact on Cu2Zn (Sn, Ge) Se4 thin-film solar cells: Towards semi-transparent solar cells. Sol. Energy.

[8] Zahoor F., Azni Zulkifli T.Z., Khanday F.A. (2020). Resistive random access memory (RRAM): An overview of materials, switching mechanism, performance, multilevel cell (MLC) storage, modeling, and applications. Nanoscale Res. Lett..

[9] Shen Z., Zhao C., Qi Y., Xu W., Liu Y., Mitrovic I.Z., Yang L., Zhao C. (2020). Advances of RRAM devices: Resistive switching mechanisms, materials and bionic synaptic application. J. Nanomater..

[10] Hong X., Loy D.J., Dananjaya P.A., Tan F., Ng C., Lew W. (2018). Oxide-based RRAM materials for neuromorphic computing. J. Mater. Sci..

[11] Park S., Kim H., Choo M., Noh J., Sheri A., Jung S., Seo K., Park J., Kim S., Lee W. RRAM-based synapse for neuromorphic system with pattern recognition function. Proceedings of the 2012 IEEE International Electron Devices Meeting.

[12] Chen Y.Y., Goux L., Clima S., Govoreanu B., Degraeve R., Kar G.S., Fantini A., Groeseneken G., Wouters D.J., Jurczak M. (2013). Endurance/Retention Trade-off on HfO2/Metal Cap 1T1R Bipolar RRAM. IEEE Trans. Electron Dev..

[13] Lee S.R., Kim Y.B., Chang M., Kim K.M., Lee C.B., Hur J.H., Park G.S., Lee M.J., Kim C.J., Chung U.I. Multi-level switching of triple-layered TaOx RRAM with excellent reliability for storage class memory. Proceedings of the 2012 IEEE Symposium on VLSI Technology (VLSIT).

[14] Guan X., Lei Z., Yu X., Lin C.H., Huang J.K., Huang C.Y., Hu L., Li F., Vinu A., Yi J. (2022). Low-Dimensional Metal-Halide Perovskites as High-Performance Materials for Memory Applications. Small.

[15] Guan X., Wan T., Hu L., Lin C.H., Yang J., Huang J.K., Huang C.Y., Shahrokhi S., Younis A., Ramadass K. (2022). A Solution-Processed All-Perovskite Memory with Dual-Band Light Response and Tri-Mode Operation. Adv. Func. Mater..

[16] Cao Q., Lu X., Wang X.R., Guan X., Wang L., Yan S., Wu T., Wang X. (2020). Nonvolatile multistates memories for high-density data storage. ACS Appl. Mater. Interfaces.

[17] Ginnaram S., Qiu J.T., Maikap S. (2020). Role of the Hf/Si Interfacial Layer on the High Performance of MoS2-Based Conductive Bridge RAM for Artificial Synapse Application. IEEE Electron. Dev..

[18] Lim S., Kwak M., Hwang H. (2018). Improved Synaptic Behavior of CBRAM Using Internal Voltage Divider for Neuromorphic Systems. IEEE Trans. Electron. Dev..

[19] Chiang K.K., Chen J.S., Wu J.J. (2012). Aluminum electrode modulated bipolar resistive switching of Al/fuel-assisted NiO x/ITO memory devices modeled with a dual-oxygen-reservoir structure. ACS Appl. Mater. Interfaces.

[20] Yanagida T., Nagashima K., Oka K., Kanai M., Klamchuen A., Park B.H., Kawai T. (2013). Scaling effect on unipolar and bipolar resistive switching of metal oxides. Sci. Rep..

[21] Ju H., Yang M.K. (2020). Duality characteristics of bipolar and unipolar resistive switching in a Pt/SrZrO3/TiOx/Pt stack. AIP Adv..

[22] Ghalamestani S.G., Goux L., Díaz-Droguett D.E., Wouters D., Lisoni J.G. (2011). WOx resistive memory elements for scaled Flash memories. MRS OPL.

[23] Lin C.L., Tang C.C., Wu S.C., Juan P.C., Kang T.K. (2015). Impact of oxygen composition of ZnO metal-oxide on unipolar resistive switching characteristics of Al/ZnO/Al resistive RAM (RRAM). Microelectron. Eng..

[24] Jinesh K.B. (2021). The effect of the top electrode on the switching behavior of bipolar Al2O3/ZnO RRAM. Microelectron. Eng..

[25] Shen W., Dittmann R., Breuer U., Waser R. (2008). Improved endurance behavior of resistive switching in (Ba, Sr) TiO 3 thin films with W top electrode. Appl. Phys. Lett..

[26] Gao B., Yu S., Xu N., Liu L.F., Sun B., Liu X.Y., Han R.Q., Kang J.F., Yu B., Wang Y.Y. Oxide-based RRAM switching mechanism: A new ion-transport-recombination model. Proceedings of the 2008 IEEE International Electron Devices Meeting.

[27] Wang H., Yan X. (2019). Overview of resistive random access memory (RRAM): Materials, filament mechanisms, performance optimization, and prospects. Rapid Res. Lett..

[28] Kim K.Y., Shim E.L., Choi Y.J. (2016). Fabrication of transparent AZO/ZnO/ITO resistive random access memory devices and their ZnO active layer deposition temperature-dependent switching characteristics. J. Nanosci. Nanotechnol..

[29] Yang P.J., Jou S., Chiu C.C. (2014). Bipolar resistive switching in transparent AZO/SiOx/ITO devices. Jpn. J. Appl. Phys..

[30] Yao J., Lin J., Dai Y., Ruan G., Yan Z., Li L., Zhong L., Natelson D., Tour J.M. (2012). Highly transparent nonvolatile resistive memory devices from silicon oxide and graphene. Nat. Commun..

[31] Thomas G. (1997). Invisible circuits. Nature.

[32] Qian K., Cai G., Nguyen V.C., Chen T., Lee P.S. (2016). Direct observation of conducting filaments in tungsten oxide based transparent resistive switching memory. ACS Appl. Mater. Interfaces.

[33] Bersuker G., Gilmer D.C., Veksler D., Yum J., Park H., Lian S., Vandelli L., Padovani A., McKenna K., Shluger A. Metal oxide RRAM switching mechanism based on conductive filament microscopic properties. Proceedings of the IEEE International Electron Devices Meeting.

