# Supplementary material for: Bipolar Switching Characteristics of Transparent WOX-Based RRAM for Synaptic Application and Neuromorphic Engineering
Source: Materials (Basel). 2022 Oct 15;15(20):7185. doi: 10.3390/ma15207185 (PMC9605663; doi:10.3390/ma15207185)
Supplement: Supplementary file 1 [file materials-15-07185-s001.zip › materials-1947549-supplementary.pdf]

# Supplementary Information

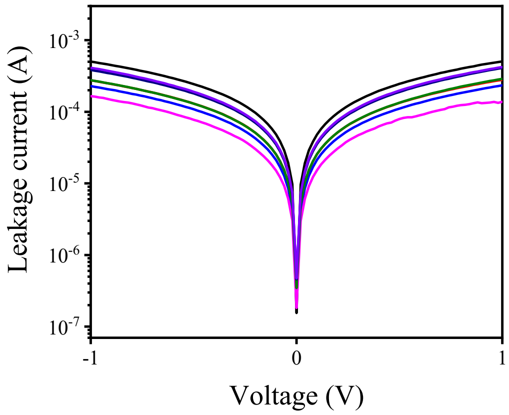


| Device | Leakage Current | |
|---|---|---|
| | $V_{read}$ = -0.2 V | $V_{read}$ = 0.2 V |
| D1 | 96.66 μA | 96.48 μA |
| D2 | 50.93 μA | 51.51 μA |
| D3 | 42.5 μA | 42.98 μA |
| D4 | 30.17 μA | 30.72 μA |
| D5 | 50.80 μA | 51.71 μA |
| D6 | 69.99 μA | 72.01 μA |
| D7 | 73.42 μA | 75.51 μA |

**Figure S1**. Device-to-device leakage current curves for positive set and negative set (different color lines indicate the measured leakage current in the seven different devices).

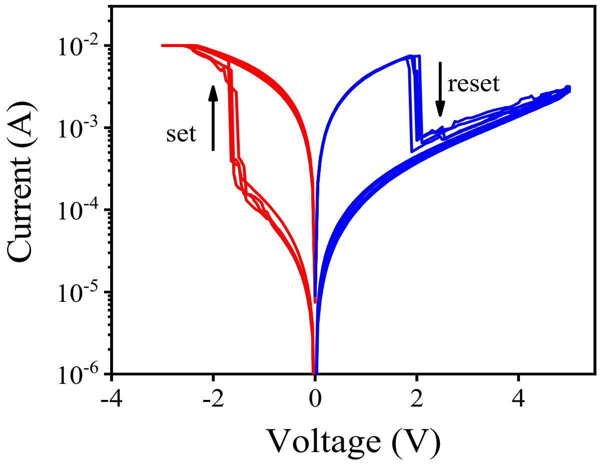


**Figure S2**. Abrupt switching curve after applying large voltage for a full reset.

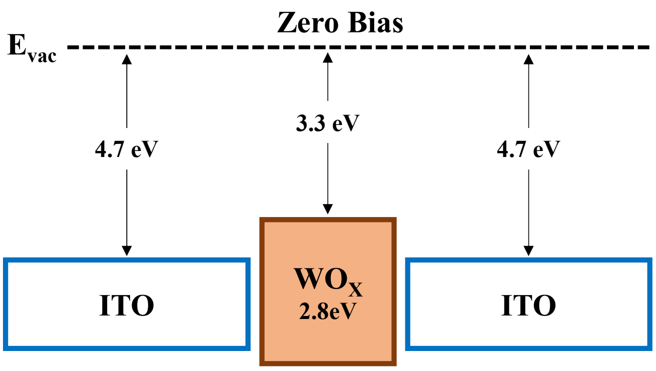


**Figure S3**. Schematic diagram of band alignment of ITO and $WO_X$.

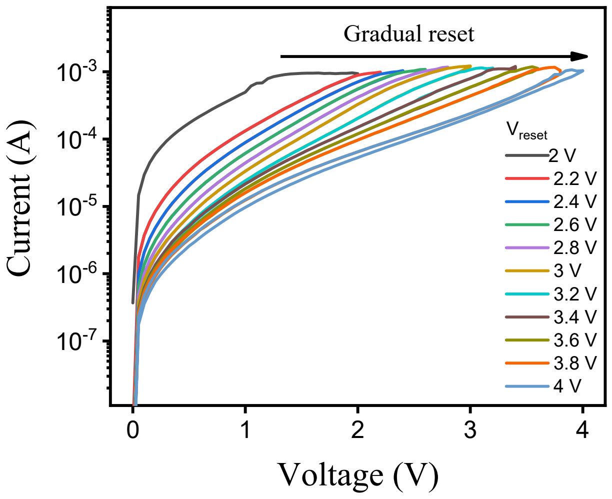


**Figure S4**. MLC by DC sweep by controlling reset voltage.
